# FRAMING CLIMATE-CHANGE INFORMATION TO PROMOTE ACTION IN OLDER ADULTS

**DOI:** 10.1093/geroni/igac059.2298

**Published:** 2022-12-20

**Authors:** Philip Lemaster

**Affiliations:** Concordia College, Moorhead, Minnesota, United States

## Abstract

Climate change poses an existential global risk, and mitigation requires population-level behavioral change. Due to their shortened time horizon, older adults may not perceive climate change as a personal risk within their lifetime and may be less likely to take action to combat climate change. Socioemotional selectivity theory (SST; Carstensen, 2006) posits that, as people age and perceive a shorter time horizon ahead, they become more focused on maintaining emotional wellbeing. This study, rooted in SST, manipulated the emotional framing of climate-change information in a sample of U.S. older adults. Participants were given EPA climate-change information localized to their state of residence and were instructed that, by taking action, the impact of climate change could be reduced (instilling hope) or that, by not taking action, the impact of climate change could be exacerbated (instilling fear). Afterward, they completed measures assessing their willingness to engage in pro-environmental behaviors and support of macro-level legislation to combat climate change. Compared to older adults who received the "hope" frame, older adults who received the "fear" frame reported greater willingness to engage in pro-environmental behaviors (p = 0.007) and greater support of legislation to combat climate change (p = 0.038), perhaps an effect of older adults' motivation to reduce the negative affective state induced by the "fear" frame. Implications of these results for catalyzing older adults toward climate action are discussed.

